# Introduction: food processing and nutritional assimilation in animals

**DOI:** 10.1098/rstb.2022.0559

**Published:** 2023-12-04

**Authors:** Myra F. Laird, Callum F. Ross, Victor Kang, Nicolai Konow

**Affiliations:** ^1^ Department of Basic and Translational Sciences, University of Pennsylvania, Philadelphia, PA 19104-6243, USA; ^2^ Department of Organismal Biology and Anatomy, University of Chicago, Chicago, IL 60637, USA; ^3^ Department of Bioengineering, Imperial College London, London SW7 2AZ, UK; ^4^ Department of Biological Sciences, University of Massachusetts, Lowell, MA 01854, USA; ^5^ UMass Movement Center, University of Massachusetts, Lowell, MA 01854, USA

**Keywords:** comparative context, performance, life history, environment, functional morphology

## Abstract

How animals process and absorb nutrients from their food is a fundamental question in biology. Despite the continuity and interaction between intraoral food processing and post-oesophageal nutritional extraction, these topics have largely been studied separately. At present, we lack a synthesis of how pre- and post-oesophageal mechanisms of food processing shape the ability of various taxa to effectively assimilate nutrients from their diet. The aim of this special issue is to catalyse a unification of these distinct approaches as a functional continuum. We highlight questions that derive from this synthesis, as well as technical advances to address these questions. At present, there is also a skew toward vertebrates in studies of feeding form–function mechanics; by including perspectives from researchers working on both vertebrates and invertebrates, we hope to stimulate integrative and comparative research on food processing and nutritional assimilation. Below, we discuss how the papers in this issue contribute to these goals in three areas: championing a functional-comparative approach, quantifying performance and emphasizing the effects of life history, and food substrate and extrinsic factors in current and future studies of oral food processing and nutritional assimilation.

This article is part of the theme issue ‘Food processing and nutritional assimilation in animals’.

## 1. Introduction

Functional morphologists and biomechanists have traditionally focused on extra-oral and intraoral aspects of feeding in vertebrates, leaving studies of the digestive system to physiologists. As a result, there is now a well-established division between these two otherwise intricately connected systems [1–4]. Such a division is counter-productive to studying evolutionary and functional processes and clearly calls for reintegration of the fields. Moreover, it has been over 20 years since the publication of the first comprehensive review of intraoral food processing in vertebrates: *Prey processing in amniotes: biomechanical and behavioral patterns of food reduction* [6]. As the title suggests, that review only included amniotes, and subsequent studies of food processing in anamniote vertebrates necessitate reconsideration of the patterns identified by Reilly *et al*. A more recent edited volume: *Feeding in vertebrates: evolution, morphology, behavior, biomechanics* [7] provided taxon-specific reviews and a starting point for broader phylogenetic comparisons of intraoral food processing across vertebrates. In this special issue, we seek to build upon those works by considering advances in food processing across a broad range of taxa and with an eye toward the function of all parts of the feeding apparatus.

In the past few decades, studies of the functional morphology of oral food processing and nutritional assimilation have been propelled forward by technological advances [8]. Cineradiography offered critical first insight into the intraoral kinematics of food processing [9–12], but the initial two-dimensional application limited its utility. Deployment of three-dimensional motion capture techniques made it possible to collect large datasets of three-dimensional jaw kinematics in primates [13–16] which, in combination with syntheses of extensive datasets on jaw muscle activity patterns [17], provided new insights into the distribution of variance in feeding behaviour within and between feeding sequences, foods and species. However, our ability to visualize and measure complex movements inside the oral cavity and pharynx has been revolutionized by development of the three-dimensional high-speed X-ray-based XROMM workflow [18]. Its technological advances, which include marker-less bone motion-reconstruction and measurements of soft tissue dynamics (strain and pennation angle changes), have accelerated the accumulation of data on how the vertebrate skull functions during feeding [19–21], how the tongue and its skeleton function during feeding [22–26], and on how muscles drive feeding apparatus function in general [27–33]. The resulting advances in our understanding of feeding significantly broaden our perspective on food processing biomechanics and support integration with physiology to explore processes occurring deep inside the organism, and not just between the jaws.

In much the same fashion, approaches to nutritional processing and assimilation have undergone significant changes in recent decades. Studies focusing on single nutritional components have given rise to multi-dimensional approaches that cover the breadth of an animal's diet and nutritional needs using a nutritional geometry framework [34]. This expansion has prompted a link to be made between nutritional processing, as framed through nutritional geometry, to, among other things, social behaviour [35], environmental change (e.g. [36]) and aspects of human health [37]. Thus, both the fields of oral and nutritional processing have undergone substantial changes that emphasize consideration of an animal's dietary breadth.

There are profound differences but also remarkable similarities between the feeding systems of vertebrate and invertebrate animals that this special issue highlights (e.g. [38–40]). While researchers for decades have used highly advanced and technical methods to study vertebrate feeding kinematics (e.g. rigid body kinematics, three-dimensional motion capture and XROMM), similar techniques have only recently been adapted to understand chewing and biting in insects [39]. Articles in this special issue focused on ants [38–40] present novel frameworks to quantitatively characterize insect mandible motion and derive bite forces across the insect phylogeny. Further development of these methods will inspire future researchers to pursue quantitative comparisons of the mechanics of feeding across a wider range of invertebrates and vertebrates.

## 2. Importance of comparative approach

Studies of feeding in general, and food processing or chewing in particular, mostly focus on mammals, including humans. This focus has led to the identification of a suite of defining traits that has been cast as characteristic of feeding function in mammals, including (i) precisely occluding dentition that is (ii) moved repetitively and rhythmically, (iii) in a set of fast and slow opening and closing phases, (iv) with jaw movements involving motion-components that are not only orthal or arcuate (up and down), but also propalinal (forward and backward) and transverse (side-to-side) [6,41]. Meanwhile, (v) the food (bolus) is transported and positioned between the teeth in between and even during individual chew cycles, via complex movements of a large, muscular and flexible tongue and the musculoskeletal system that suspends it. This suite of traits is indeed characteristic of mammal chewing, but a broad comparative analysis of intraoral feeding behaviours across gnathostomes (jawed vertebrates) reveals that most of these traits have evolutionary origins deeper in the phylogeny of jaw-bearing vertebrates, and hence may be ancestral to mammals, or have evolved in parallel in other clades.

Recent work including several papers in this issue provide some compelling examples, as they relate to the traits listed above:

(i) Some species of carp (teleost fishes) shear and grind food between teeth on the pharyngeal jaws and a pad on the base of the cranium, resembling intraoral food processing in ungulates [42]. Though clearly not homologous, the convergent evolution of precise tooth occlusion suggests that the evolutionary drivers of mastication-like food processing are common to taxa belonging to different gnathostome clades. Exactly how widespread precise occlusion is across vertebrates will require broad functional-comparative studies to determine [43]
(ii) Highly rhythmic jaw movements during chewing were once argued to be a hallmark of mammals, linked to proprioceptive specializations and highly precise occlusion between upper and lower teeth [44]. However, similar rhythmicity has been shown to also characterize chewing in many bony fishes [45], and in this issue, similar rhythmicity is also reported in basal sarcopterygians including lungfishes, and in many species of salamanders [46]. The discovery that rhythmic chewing may be both ancestral to, and the prevalent condition in, gnathostome vertebrates (with a possible exception being lepidosaurs) in turn raises questions about the role of proprioception in maintaining chew cycle rhythmicity across vertebrates, because—with one dubious exception [47]—only tetrapods have so far been shown to have muscle spindles in their jaw muscles [45]. These results emphasize the need for further study of the interplay and coevolution of chewing rhythmicity, dental occlusion and sensory feedback from the oral cavity in vertebrates.
(iii) Gape cycles involving fast and slow opening and closing phases of the jaw movement have been championed as characteristic of tetrapod feeding since Bramble and Wake [48]. Capitalizing on the high precision and accuracy of biplanar videofluoroscopy for analysing mandibular jaw movements, Richards *et al.* [46] sampled gnathostome taxa, from elasmobranch rays to salamanders, to demonstrate that having a chew cycle divided into four distinct phases may in fact precede the evolution of tetrapods, and possibly be as ancient as gnathostomes themselves. Whether these four phases are encoded in the motor patterns of gnathostomes, and/or emerge from interaction between the jaws, the tongue, the food and the ambient environment remains to be determined.
(iv) X-ray-based studies more recently provided several examples of how jaw movements in aquatic-feeding anamniotes (fishes and salamanders) are not simply orthal or arcuate (up and down; like ‘Pac-man’) but also involve translational components, with either propalinal and/or transverse motions [46], as for instance in the freshwater stingray [49] and the salamander *Siren intermedia* [50]. Based on morphological analyses of their jaw joints, it has also been suggested that some dinosaurs translated their jaws side-to-side as they chewed [51].
(v) Salamanders are key transitional taxa from aquatic-feeding anamniotes to tetrapods, and it has often been proposed that they—uniquely among tetrapods—do not chew their food but instead transport it directly to the oesophagus for swallowing (e.g. [52]). However, by using precise X-ray-based approaches to discriminate intraoral food movements and tongue behaviours, Spence *et al*. [33] show that at least in the Mexican salamander (Axolotl; *Ambystoma mexicanum*) chewing does in fact occur. Surprisingly, Spence *et al*. also show that the tongue of this ancestral tetrapod functions during transport cycles to move the food cheek-wards, towards the hard surfaces used to process it intraorally [6]. This finding once again suggests a more ancestral origin of complex intraoral food handling than previously established.

The handful of findings presented above suggest that many of the traits that have been cast as characteristic of mammal chewing are also seen in more basal vertebrates. Some of these traits appear to have evolutionary origins deeper in vertebrate phylogeny than the origin of mammals and have therefore probably been retained in mammals (e.g. a four-phase gape cycle). Others are likely to be convergent (e.g. occlusal functional wear of carp teeth to improve food processing, use of the tongue to position the food between the teeth during chewing in salamanders). Regardless of whether these traits are ancestral or convergent, their presence in combinations different from those seen in mammals suggests that functional coupling between traits—e.g. between using the tongue to move food transversely within the oral cavity, precise occlusion between upper and lower teeth, rhythmic jaw movements, a four-phase gape cycle—is lineage-specific and evolutionarily malleable. For example, since many bony fishes and salamanders move their jaws in transverse, grinding fashions, this trait does not necessarily depend on the evolutionary differentiation of the ancestral adductor mandibulae complex into distinct temporalis, masseteric and pterygoid muscles, but may represent a dietary signal (herbivory). Assuming poorly substantiated links between morphology and behaviour, or between different aspects of feeding behaviour, can also introduce errors in hypotheses about the way that extinct taxa fed (see [46]).

## 3. Importance of understanding different performance metrics

Determining the performance of any biological system invariably involves an assessment of function, but animals have a range of performance capacities that can define a space within which all activities occur [53]. This space provides an approach for comparison between individuals and can vary depending on factors such as environment, substrate, animal or individual [53]. Evolutionarily, measures of performance provide an assessment of how the morphology of an organism reflects natural selection as these measures relate phenotypes to ecology [54]. For feeding, performance metrics commonly include measures such as volume, duration, force, distance, and speed. Performance capacities measured by these common metrics are readily applicable to oral processing and can be used to define a performance space within which comparisons can be made across animals. However, we suggest that such measures may be less suitable to assess performance during nutritional assimilation and questions remain as to the overall importance of specific performance measures during pre- and post-oesophageal food processing.

Oral feeding performance is defined in this issue through a range of conventional measures. Püffel *et al*. [40] define mandibular performance in feeding ants using bite force production, and Richter & Economo [38] discuss performance though food uptake rates and feeding duration. Similarly, Bels *et al*. [55] highlight measures of performance for food transport in lizards involving measures of prey size per cycle, gape, speed of transport and number of cycles. Laird *et al*. [32], Olson *et al*. [26], Richard *et al*. [46] Spence *et al.* [33] and Stilson *et al*. [21] all assess feeding performance through jaw and tongue kinematics, as well as muscle performance (length-change), whereas Panagiotopoulou *et al*. [56] use bone strain. Strain patterns and changes in muscle architecture are proxies for force production, energetic costs and feeding duration; although we note that many of the relationships between measures of performance remain tenuous (with noted exceptions, e.g. [57,58]). Energy costs provide an important performance metric for the locomotor system, but the role of energy costs in feeding system morphology and adaptations is debated and largely untested [13,59,60]. Wall *et al*. [61] actually measured the energetic costs of feeding in a wide range of primates, and their data provide a fundamental baseline for testing hypotheses about the importance of energetic efficiency in driving the evolution of intraoral feeding behaviours in mammals, in addition to evidence previously presented for feeding in select animals [62–64]. While straightforward, measures of feeding performance have only been obtained from select vertebrates and invertebrates, leaving outstanding questions about how these measures relate to each other and vary in correspondence with changes in morphology, ecology and behaviour.

Further down the digestive tract, performance can also be assessed through a diverse set of measures. Conventional measures of performance such as digestibility and fecal particle size are relevant to nutritional processing (e.g. [65,66]), but assessments of performance often extend beyond these measures. For example, Li *et al*. [67] define swallowing performance as the safe and efficient bolus transport from the oral cavity to the oesophagus, whereas Clauss *et al*. [68] explore the relationships between chewing and digestive efficiency through a *washing mechanism*. Beale *et al*. [36] and Raubenheimer *et al*. [69] both assess performance through nutritional and secondary compound intake. This divergence in performance measures reflects the current separation between oral processing and nutritional assimilation studies. The degree of oral processing has well-established links to the amount of nutritional assimilation occuring in the digestive system [70], and nutritional needs can dictate food choice and oral processing. A reunification of these study areas will improve our understanding of how performance measures relate to each other and their roles in different parts of the digestive tract.

Thus, we suggest that part of the disjunct between oral food processing and nutritional assimilation is a lack of comparable performance measures, and/or a lack of understanding of how different measures are related. Many of the basic metrics of food breakdown, speed and capacity have not been tracked throughout the digestive tract, and methods such as XROMM have strong potential for collection of such data. Ambiguity in performance terms has been implicated as an issue for clinical approaches to oral processing in humans [71,72]. Further, we lack assessments of the importance of specific performance measures and how they relate to one another. Outside of using a straw, the ability for adult humans to produce suction is far less important for acquiring food relative to a suction-feeding fish, and the relationship between bite force and the energetic costs of chewing, though intuitively obvious, remains incompletely understood. Similarly, performance variables may be contextual or clade-specific. Oral processing speed or volume may be important for certain environments or substrates but inconsequential in others. We believe that only by bridging these gaps in our understanding of performance variables, how they relate to each other, and their relative importance can we achieve a comprehensive understanding of the integrated evolution of intraoral processing and nutritional assimilation.

## 4. The effects of substrate and extrinsic factors

Food processing and nutritional assimilation are highly dependent on extrinsic factors, including the animal's environment and the food substrate. Evolutionarily, this interdependency is perhaps best illustrated by the transition from feeding in water to feeding on land, which involves accompanying changes in the mechanics of the feeding system as it shifts from operating in water to air, as well as in the nutritional needs of the animal and in the availability of food (discussed in [73]). It is well recognized that, fundamentally, these changes are dictated by extrinsic changes in the physics of the environment (drag, gravity, buoyancy and viscosity; see [74] for a recent discussion). Factors affecting the food bolus are sourced from the external environment and from inside the oropharyngeal cavity. The fact that microbes represent an important food source—not only those found in the environment in ‘farming’ invertebrates [75] or ‘detritivorous’ fish [76], but also those living in the digestive tract of many mammals that have evolved adaptations for their harvest [68]—is not reflected in traditional trophic niche concepts of faunivory–omnivory–herbivory. Moreover, in the context of major environmental transitions, there are likely emergent intrinsic properties, for instance resulting from interactions between the environment in which feeding occurs and the substratum that is being fed upon, and such emergent properties are yet to be made explicit. For instance, as organisms invade land, drag on the feeding system from the surrounding aqueous medium subsides, and meanwhile there is also an associated collapse of the buccal volume that was distended by water. This environment versus food-substratum interaction likely results in new, functionally relevant shear forces, exerted between the food and the reshaped intraoral structures. Similar interactions can be hypothesized for animals that alter their dietary niche, e.g. through seasonal exploitation of fallback foods, in animals that drastically alter their posture during feeding, such as for instance behavioural changes including hanging upside-down during mastication, as seen in bats [77], due to postural changes across evolution, as explored by Li *et al.* [67], or due to ontogenetic changes such as for instance chewing and swallowing before and after laryngeal descent, as encountered by most mammals, and to a particularly dramatic extent by ungulate males [78]. Associated implications for optimal performance could include changes in the pattern of activation of muscles driving the feeding system, novel interactions between contracting muscles and elastic action of the tendons that connect them to the bones they move, or a combination thereof.

Similar changes more intrinsic to the organism may result from the physical properties of the food substrate. Biological anthropologists have led the way in understanding how food material properties (toughness, hardness or strain to fracture) influence feeding system function (muscle activation, force production and jaw motion speed) [17,27,79,80]. However, studies relating food material properties to oral processing in vertebrates are limited outside of primates (but see, [44,77,81]). In this special issue, Stilson *et al*. [21], Laird *et al*. [32], Wall *et al*. [61] and Panagiotopoulou *et al*. [56] all explore effects of varying food substrates during oral processing. Despite the importance of understanding the physics of a food substrate, this element of oral processing has thus far had a relatively limited effect on linking diet and morphology within primates [14]. In the case of invertebrates, there is a substantial body of work on the relationship between insect feeding and plant material properties, particularly in the field of crop protection (e.g. [82–84]). One of the challenges of studying the effect of plant substrates on feeding is the extreme heterogeneity of plant leaves, which makes quantifying their physical properties particularly difficult. In this issue, Püffel *et al*. [40] use a synthetic substrate to standardize the material properties in order to investigate underlying relationships between feeding system function (e.g. cutting force and speed) and mandible size and wear. However, the links between food processing and nutritional assimilation in the gastrointestinal tract, as they relate to food material properties, have yet to be made explicit for any given vertebrate or invertebrate taxon.

Understanding how food processing and nutritional assimilation change with variation in substrate and the environment is also particularly relevant to understanding the influence of environmental change. Increasing climate variability, aridification and warming are factors that have the potential to influence the entire pre- and post-oesophageal system as the ecological profiles of the world's animals change and their food resources shift. Climate change, for example, is likely to increase the threat of herbivorous insects in agriculture and forestry. As regulations against unsubstantiated chemical insecticide use are becoming stricter, growers need alternative options to protect their crops, such as identifying plant features—both chemical and physical—that deter insect herbivory. However, little is known about the fundamental mechanisms behind insect feeding, including how herbivorous insects move their mandibles for cutting, what factors influence the cutting force, and the range of bite forces across different insects. Several papers in this special issue address some of these questions by quantifying insect mandible motion and deriving bite forces across the insect phylogeny [39,40]. Such insights can inspire further work to understand insect feeding systems and to help researchers identify suitably resistant crops or to inspire new environmentally friendly methods for crop protection. Thus, an integrated understanding of the effects of environmental change on animals is reliant upon the reconciliation of knowledge about food processing and nutritional assimilation.

## 5. Life history

By necessity, intraoral processing and nutritional assimilation persist across the lifetime of the organism and must remain flexible to life-history changes associated with maturation, weaning and ageing. Life-history changes in oral processing have focused on the mammalian transition from suckling to chewing. During this transition in mammals, oral processing undergoes a monumental shift from a fluid substrate to solid or semisolid foods, resulting in changes in muscle activation patterns, jaw loading, tongue movements and swallowing [71,72,85,86]. However, animals other than mammals also undergo developmental transitions necessitating changes in oral processing, and there is a distinct lack of studies exploring these transitions. Many salamanders, for example, develop from an aquatic juvenile phase to land-living, air-breathing adults. Insects are highly diverse in the extent to which they undergo changes in nutritional assimilation during development: some, like grasshopper nymphs, are akin to smaller versions of the adult, and use the same feeding systems; others, such as ants and mosquitoes, metamorphosize and change their feeding apparatus, food preferences and behaviour (see [87] for more examples). Changes in nutritional assimilation have been studied in relation to ageing and notably calorie restriction [88], but additional work is needed to explore shifts in nutritional geometry with intraoral processing transitions during specific life-history events, such as weaning. Thus, there is a need for comparative work outside of mammals on oral processing transitions early in life history and for a better understanding of how nutritional assimilation changes in response to these events.

Most of the studies in this issue examine food processing and nutritional assimilation across short time-scales. While short-term studies provide valuable insights, there is a need for research across longer time-scales to understand how these systems respond to ageing, and in turn, how food processing and nutritional assimilation impact overall health, growth and disease prevention over extended periods (reviewed in [71,72,86]).

Much of the work on ageing in oral processing relates to dysphagia, or difficulty swallowing, in humans and select model systems such as the pig. We suggest that comparative studies examining late-in-life changes in oral processing in a variety of taxa can inform the origins and mechanisms underlying these changes, with potential implications for questions in human health. Techniques such as XROMM provide unique opportunities, especially in short-lived organisms, of cradle-to-grave processes in animal feeding function.

## 6. Concluding remarks

It is within the framework laid out above that we acknowledge three shortcomings and proposed future directions of study highlighted by this special issue.

The first is that we are still limited in the number of studies directly connecting intraoral food processing and nutritional assimilation. Instead, many studies can be divided into one of these areas despite the close relationship between food processing and nutritional assimilation [89]. One consideration is the complexity within each of these categories highlighted by studies in this issue (e.g. [68]). The second future direction is that there are few comparisons of vertebrate and invertebrate food processing and nutritional assimilation. Richter & Economo [38] make important comparisons between the anatomy and function of the feeding system in vertebrates and invertebrates, and it's clear that performance metrics such as bite force and jaw kinematics are important variables in both taxa (see [40] and [39]). Comparisons of vertebrate and invertebrate animal feeding systems will inform our understanding of food processing in evolutionary, performance and environmental contexts. Third, we note the need for parallel studies merging invertebrate food and nutritional processing. Invertebrate nutritional assimilation has been well studied (e.g. [90]), but unlike vertebrate studies, oral and intraoral feeding studies of invertebrate taxa are at a relatively nascent stage, whereas similar to vertebrate studies, there are few specifically linking oral food processing to nutritional extraction.

In sum, the papers in this special issue highlight how intraoral and post-oesophageal processes in feeding are interrelated, vary across invertebrates and vertebrates and require broad integrative and comparative analyses. We propose the consideration of collaborations that bring together studies of diverse animal systems addressing performance criteria and response variables, study the roles of substrate, extrinsic factors and life history, and compare these broadly within a phylogenetic framework. Such analyses have the potential to both reveal generalized themes and key differences in feeding, from oral to intestinal, across the animal tree of life.

## Data Availability

No new data were used to write this article.
